# Performance Evaluation of Jute/Glass-Fiber-Reinforced Polybutylene Succinate (PBS) Hybrid Composites with Different Layering Configurations

**DOI:** 10.3390/ma15031055

**Published:** 2022-01-29

**Authors:** Muhammad Usman Ghani, Amna Siddique, Kahsay Gebresilassie Abraha, Lan Yao, Wei Li, Muhammad Qamar Khan, Ick-Soo Kim

**Affiliations:** 1 College of Textiles, Donghua University, Shanghai 201620, China; ghani7480@gmail.com (M.U.G.); kahsaymam6@gmail.com (K.G.A.); lanyao@dhu.edu.cn (L.Y.); 2 Center for Civil Aviation Composites, Shanghai 201620, China; 3 Key Lab of Textile Science & Technology, Ministry of Education, Shanghai 201620, China; 4 School of Engineering and Technology, National Textile University, Faisalabad 38000, Pakistan; amnasiddique104@hotmail.com; 5 Department of Textile Engineering, College of Engineering and Technology, Aksum University, Aksum P.O. Box 1010, Tigray, Ethiopia; 6 Nanotechnology Research Group, Department of Textile and Clothing, Faculty of Engineering and Technology, National Textile University Karachi Campus, Industrial Area Korangi, Karachi 74900, Pakistan; 7 Division of Frontier Fiber, Institute of Fiber Engineering, Interdisciplinary Cluster for Cutting Edge Research (ICCER), Faculty of Textile Sciences, Shinshu University, Nagano 386-8567, Japan

**Keywords:** jute/glass fabric hybrid composites, tensile properties, flexural properties, thermal properties, water absorption properties

## Abstract

The hybridization of natural and synthetic fibers leads to composites’ optimum mechanical properties. In this study, an attempt was made to study the effect of the stacking sequence on PBS-based Glass-Jute (GJ) hybrid composites. Six types of hybrid composite, each containing five different layers of jute and glass fabric, were manufactured by the compression molding method. Mechanical properties, such as tensile, flexural, and impact resistance were studied and analyzed in detail. The surface characterization of the composites was performed through scanning electron microscopic images. The moisture absorption properties were also investigated by immersing the composites in distilled water for one week at ambient temperature. The TGA test was conducted to study their thermal properties. The experimental results showed that the stacking sequence of the fiber layers has a significant effect on the overall performance of GJ hybrid composites. Among the hybrid GJ composites, composites with glass fiber layers on their outer surfaces showed optimum mechanical, thermal, and water resistance properties.

## 1. Introduction

Due to increased environmental and renewable green resource concerns over the past few decades, natural-fiber-reinforced polymer composites have received substantial interest from researchers as the next stage of composite products [1]. Notwithstanding the benefits of NFRP composites over synthetic fiber composites, such as higher ecological performance, lower density, reusability, and renewability, NERP composites have significant limitations, such as low mechanical performance and the restricted compatibility of their matrix and natural reinforcement [2]. Another drawback of natural fiber composites is their weak resistance to moisture absorption, which makes them less attractive. Due to these limitations, natural fiber composites can only be applied in structural components with particularly low mechanical demands [3]. In recent decades, various modification methods have been established to improve the performance of natural fiber composites. One of the most promising approaches involves mixing natural and synthetic fibers in a single composite material. The benefits of each fiber type may be merged into a hybrid composite utilizing this method [4].

Among natural fibers, jute is a common plant; hence, using this widely accessible and economically viable natural resource in composite materials for multiple purposes has a favorable influence on national and worldwide sustainability [5]. There are, however, significant restrictions in terms of mechanical characteristics and water resistance when assessing hard service conditions and structural applications [6,7,8,9]. Therefore, in order to overcome the above-mentioned issues, jute fibers must be hybridized with a synthetic reinforcement that can accommodate the aforementioned limitations due to their better mechanical properties compared to natural fibers [10]. 

Currently, glass fibers are used as the dominant synthetic fibers in hybrid composites owing to their high mechanical performance, high density, and low cost. It has been reported that the hybridization of glass fibers with jute fibers can result in the improved mechanical performance of GJ hybrid composites [11,12,13]. The addition of glass fibers improved the environmental durability of natural fiber composites [14]. The incorporation of sugar palm and glass fiber into the TPU matrix resulted in increased tensile and impact strength with increased sugar palm fiber [15]. The tensile properties of various hybrid composites were enhanced with glass fiber contents [16].The effects of glass hybridization on sisal-reinforced polypropylene (PP) composites’ mechanical properties were studied, and it was observed that hybridization improved the mechanical properties and thermal stability of the PP composites. The temperature of the thermal decomposition of the composites increased with increased glass fiber content [17].

The effects of the stacking sequence on GJ hybrid composites have been studied by several researchers [18,19,20]. It has been shown that the mechanical characteristics of hybrid composites reinforced with jute, kenaf, and E-glass fiber reinforcements are improved when the layers are stacked in a certain order, as described by Sanjay et al. [21]. Furthermore, it was reported that glass/kenaf reinforcement layers used as skin and jute reinforcement layers used as the core exhibited optimum performance compared with other hybrid laminates. Among different hybrid composites, it was found that glass/kenaf reinforcement layers as skin and jute reinforcement layers as core performed the best [22]. Ramesh et al. [23] produced composites with sisal, jute, and glass fiber. Tensile and flexural tests were conducted on the composites. The authors found that sisal fiber composite has better tensile strength than jute fiber composite, but both have less tensile strength compared to glass fiber composite. In a study of jute/glass composites, Ahmed and Vijayarangan [24] discovered that placing glass fibers at the ends enhanced flexural strength in comparison to composites with intercalated jute and glass layers. Selver et al. [20] investigated the tensile, flexural, and thermomechanical characteristics of flax/glass and jute/glass hybrid composites. The composites were made using the vacuum infusion process, and the flexural strength was found to be enhanced when glass fiber was added to the outer layers (“glass/flax/glass” or “glass/jute/glass”), as well as when the jute and flax fiber percentages were increased.

Unlike thermoset polymers, thermoplastic polymers may be melted and recycled several times. Although thermoplastics are very lightweight and simple to produce, they have a poorer heat resistance than other materials. The thermoplastic polymer, PBS, was employed as a matrix in this study. Four types of thermoplastics are being used these days, based on their origin and biodegradability: polylactic acid and bio-polypropylene, both biobased but not biodegradable. Green polypropylene and polyethylene are examples of fossil-based and non-biodegradable plastic polymers. Another biodegradable thermoplastic matrix material, made from fossil-based components yet biodegradable, falls into the fourth group [25]. Compared to commonly used biodegradable polymers, PBS has better mechanical and thermal characteristics and processability [26,27]. A synthetic aliphatic polyester, 1,4-butanediol, and succinic acid are mixed to create PBS. PBS has a propensity to hydrolyze and is biodegradable [28]. However, to the best of our knowledge, no studies have been conducted on the use of PBS as a matrix in GJ hybrid composites. 

In this study, PBS, a thermoplastic polymer, was used as a matrix and hybrid composites (woven jute fabric/glass fabric hybrid) were fabricated and investigated for mechanical, water absorption and thermal properties. Moreover, the effect of glass hybridization and the effect of the layering pattern on the mechanical properties of hybrid composites was studied.

## 2. Experimental Design

### 2.1. Materials

Plain woven jute fabrics were purchased from Plain Textiles, China. Plain woven glass fabrics were supplied by Dongguan Kaixili Material Co., Guangzhou, China. The biodegradable PBS pellets (#1001) with a melting temperature of 90 °C were bought from Changshu Jiafa Chemical Co. Ltd., Suzhou, China. The areal density of jute fabric and glass fabric was 300 g/m^2^, 450 g/m^2^, whereas density of PBS was 1.26 g/cm^3^. Fabric properties are given in Table 1. All the chemicals and reagents were used without any further rectification.

### 2.2. Fabrication of Poly (Butylene Succinate) (PBS) Film

Poly (butylene succinate) (PBS) granules were dried in an oven for four hours and subsequently processed into films with a thickness of 0.2 (±0.01) mm at 150 °C by introducing a pressure of 10 MPa over 10 min using a compression molding machine via frame mold with dimensions of 260 × 260 mm^2^. The PBS films were removed and cooled down at room temperature. Subsequently, these films were kept at room temperature in the laboratory.

### 2.3. Fabrication of Composite Panels

**Figure 1:**
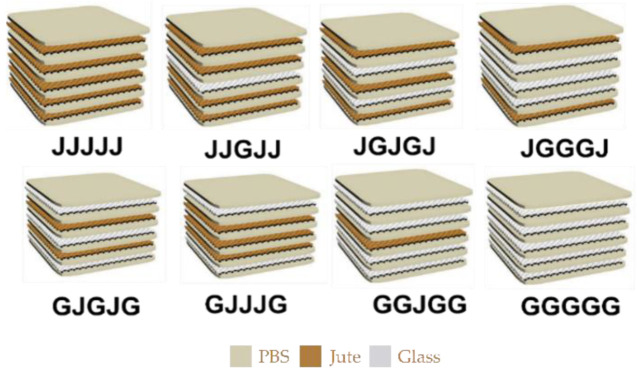
Stacking sequences of fabric plies.

The jute fabric was dried by placing it in a vacuum oven at 60 °C for 24 h. The PBS films, jute fabric, and glass fabric were cut into sizes of 260 × 260 mm. Both fabric sheets were cut in the warp direction of the yarns. Six groups of hybrid composites were produced with five plies by altering jute and glass fabrics, as shown in Table 2 and Figure 1. Composites consisting of only jute fabric and glass fabric were prepared for comparison purposes to analyze the hybridization and stacking sequences’ effect on the mechanical properties. Fabric sheets were arranged by sandwiching five layers of fabrics between seven layers of PBS for each composite. Each layer of jute and glass fiber represents 20% of the available fiber volume fraction.

**Figure 2:**
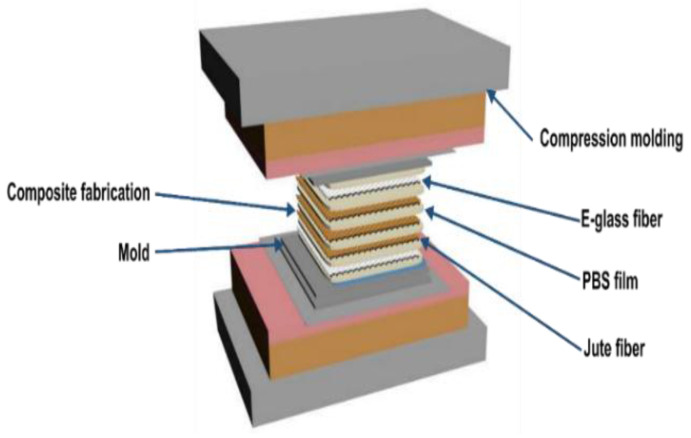
Fabrication of composite panels.

The total fiber content in the composite was kept constant at 30% by volume. Finally, the prepared structures were pressed in a stainless-steel mold with a thickness of 2.5 mm at a pressure of 12 MPa at 150 °C for 10 min in the compression molding machine to obtain composite panels, as shown in Figure 2. 

### 2.4. Tensile Testing

Tensile testing samples with a length of 250 mm and a width of 25 mm were cut out of the composite panels in the warp direction by a water jet wheel saw and were made to exact size with emery paper. The specimens were conditioned before testing at 23 ± 1 °C and 50 ± 1% R.H. for 24 h. Glass-reinforced plastic tabs were linked to the samples to ensure proper grip and failure in gauge length. The composite tensile properties were measured under ASTM D3039 and a loading rate of 2 mm/min was used for testing. The test was conducted by introducing tension until fracture occurred. Three identical samples were tested for each stacking sequence, and the final results were obtained. 

### 2.5. Flexural Testing

Flexural testing, as per ASTM D 790, was carried out on the same tensile testing machine. Samples with a length of 127 mm and a width of 12.7 mm were cut from the composite panels in the warp direction and were finished to the exact size by emery cloth. The specimens were conditioned before testing at 23 ± 1 °C and 50 ± 1% R.H., for 24 h. Samples were loaded with a suggested span-to-depth ratio of 16:1 at three bending points. The test was performed with a load cell of 30 kN at a load rate of 2 mm/min on the same machine. Three identical samples were tested for each stacking sequence, and the average results were obtained. The flexural strength and modulus were calculated by using Equations (1) and (2), respectively.

(1) $$\sigma =\frac{3PL}{2bh2}$$

where *σ* is the stress at the surface of the sample (MPa), *P* is the applied force (N), *L* is the support span (mm), *b* is the width of the sample (mm), and *h* is the thickness of the sample (mm).

(2) $$Ef=\frac{mL3}{4bh3}$$

where *Ef* is flexural modulus of elasticity (MPa), *L* is support span (mm), *b* is width of the sample (mm), *h* is thickness of the sample (mm), and *m* is the slope of the force-deflection curve. 

### 2.6. Impact Strength Testing

An impact strength test was performed on a digital Izod impact tester. The ISO 180 was followed to prepare the samples for testing. Samples were cut from composite panels through a water jut cutter and then finished to 80 × 10 mm^2^ with an emery cloth. The hammer’s speed was 5.5 m/s. The specimens were conditioned before testing at 23 ± 1 °C and 50 ± 1% R.H. for 24 h. The specimens were placed vertically through a clamp and broken by a single pendulum swing. The impact occurred on the specimen’s notched side. Five samples were tested at 30 °C for each case. 

### 2.7. Scanning Electron Microscopy (SEM)

A scanning electron microscope (SEM, TM 3000, Hitachi, Japan) was used to examine the morphological structures of the composites. For this purpose, thin and uniform sections were cut out from fractured tensile samples and attached using conductive silver paint on an aluminum stub, then sputter-coated through gold before the morphological examination. The images of the specimens were obtained with a magnification of ×50.

### 2.8. Water Absorption Test

A water absorption test was performed as per ASTM standard ASTM D570. The samples were first dried in the oven and cooled, and then their initial weights were measured. The samples were submerged in water at room temperature to calculate initial water absorption (%) for 24 h and dried with a lint-free cloth before final weight measurement. The weight gain percentages of the samples were measured for a week every 24 h by Equation (3).

(3) $$W_{e}\left(t\right)=100\times \left(\frac{W_{t}-W_{0}}{W_{0}}\right)$$

Where $W_{e}$s the relative weight change, $W_{t}$ is the final weight,$\;W_{0}$ is the initial weight at *t* = 0, and tis the soaking time. The thickness swelling percentage was assessed by using Equation (4).

(4) $$T_{re}\left(t\right)=100\times \left(\frac{T_{t}-T_{0}}{T_{0}}\right)$$

where $T_{re}$ is the percentage of thickness swelling, $T_{t}$ is the thickness at time *t*, and $T_{0}$ is the initial thickness at *t* = 0. 

### 2.9. Thermal Studies

TGAs (thermo-gravimetric analyses) were conducted on a TA instrument (TGA 4000, PerkinElmer, Waltham, MA, USA) under N_2_. Temperature analyses ranging from 30 °C to 600 °C at a heating rate of 15 °C/min were conducted. The sample quantity used was about 5.0 mg.

## 3. Results and Discussion

### 3.1. Tensile Properties

**Figure 3:**
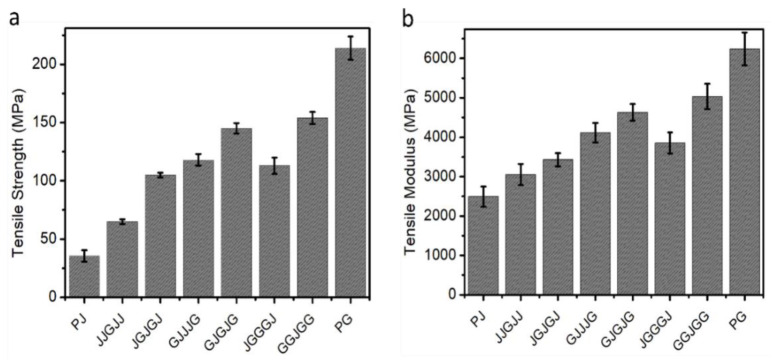
(**a**) Tensile strength and (**b**) tensile modulus of different samples.

Figure 3 shows the tensile strength and modulus of the pure jute, pure glass, and six hybrid composites with different stacking sequences, which were highly dependent on the tensile strength and modulus of the fibers. The tensile strength and modulus results showed that the tensile strength of jute composite was lower than that of the hybrids and of the pure glass composite. The high mechanical strength of the glass fiber resulted in a positive hybrid effect with the enhancement of the tensile properties. The increase in tensile strength was primarily related to the number of glass layers in the composites. The tensile strength and modulus of composite JJGJJ were observed to be 66.6% higher than the those of the pure jute composite. As the number of glass fiber layers increased, the tensile properties of the composites further improved, which showed the direct relation of the tensile strength to the number of glass plies in the composites. 

It can be observed that the stacking sequence has a significant effect on the properties of hybrid composites. The tensile strength and modulus were observed to be higher when the glass fibers were used as outer layers; this was observed in the JGJGJ, GJJJG, JGGGJ, GJGJG, and GGJGG composites. When comparing hybrid composites with the same number of jute and glass fiber layers but different stacking sequences, it was observed that GJJJG showed an increase of 12% and 20% in tensile strength and modulus, respectively, relative to the JGJGJ hybrid composite. Likewise, the hybrid composite GJGJG had 28% and 20% higher tensile strength and modulus, respectively, compared to the JGGGJ hybrid composites. It is certain that the glass fibers were more efficient at resisting heavy loads before transferring these loads to the natural fibers. In the specimens with natural fibers in the outer layer, the natural fibers could not carry heavy loads, which caused the synthetic fibers (glass fibers) to withstand most of the load or stress. The higher tensile strength of hybrid composites is attributed to stronger and stiffer glass fibers. As jute fibers possess a lower strain at break than glass fibers, the breaking of the former fibers was responsible for determining the composite’s failure. Glass fiber failure transferred high stress to the less durable jute fibers, eventually leading to the failure of the composite.

### 3.2. Flexural Properties

**Figure 4:**
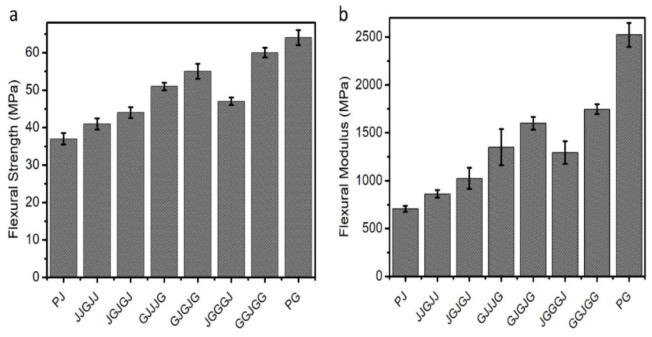
(**a**) Flexural strength (**b**) Flexuralmodulus of different samples.

Flexural characteristics reflect a material’s flexibility, and proper flexural strength suggests that materials are brittle and hard [29]. Using a three-point bending testing method, transverse bending testing was the most frequently used approach. Circular or rectangular cross-section rod specimens bend until fracture occurs. Table 3 and Figure 4 show the influence of different stacking arrangements on the flexural properties of the jute composite, glass composite, and hybrid composites. A similar trend to the tensile test was noted for the bending testing due to the improved flexural strength and flexural modulus using glass fiber hybridization. The pure glass composite had the highest stress to resist deformation under flexural deformation. Because the glass fibers consisted of finer fiber orientations than the natural fibers, the glass fibers withstood higher forces than the natural fibers. The fibers were efficiently involved in the stress transfer due to the excellent interfacial bonding between the glass fiber and the PBS film. According to our observations, all the hybrid composites showed significant enhancement in bending strength and modulus relative to the jute composite. In all the hybrid composites, the flexural strength and modulus fluctuated between pure glass composite (64 MPa, 2543 MPa) and pure jute composite (37 MPa, 701 MPa).

The addition of one ply of glass fiber improved the flexural strength and modulus by 10.62% and 22.96% compared to the pure jute composite. It was seen that the hybrid composite GJJJ, showed 16% and 32% higher flexural strength and modulus than composite JGJGJ, respectively. In comparison, the composite GJGJG exhibited 17% and 24% higher flexural strength and modulus, respectively, relative to the JGGGJ composite. This is related to the fact, as many researchers have reported, the use of high-strength fiber as skin improves the composite’s mechanical properties. One reason is that the high strength and modulus of woven glass fiber provided at the top and bottom layer withstand the applied load, whereas the core (jute) absorbs and distributes the loads uniformly [30,31]. The other reason for the difference between the two stacking sequences is that glass fibers have much greater bending stiffness than natural fibers. During bending loading, the sample surface is greater than the center of the sample [32]. Subsequently, the top layer of the reinforcement layer controls the flexural strength and stiffness of the specimen. Generally, failure begins on the composites’ outer layers and extends to the inside layers during flexural testing. As a consequence, the flexural strength or force of a composite is proportional to the bending stiffness of the outer layers. These results are consistent with those of previous studies focusing on the effect of alternate stacking sequences on composite performance [20].

Our observations indicated no breakage of specimens during testing. There was no matrix failure, and no evidence was found for jute or the glass ply delamination. The failure occurred due to the bending of the specimens at the peak load. This was due to the fact that the top fiber plies and the PBS matrix absorbed most of the force applied during bending, and the shear created between the top and bottom fiber plies as well as the matrix resisted the external force and transferred the force to the internal plies, which resulted in the enhanced flexural strength and modulus of the specimens.

### 3.3. Izod Impact Test

**Figure 5:**
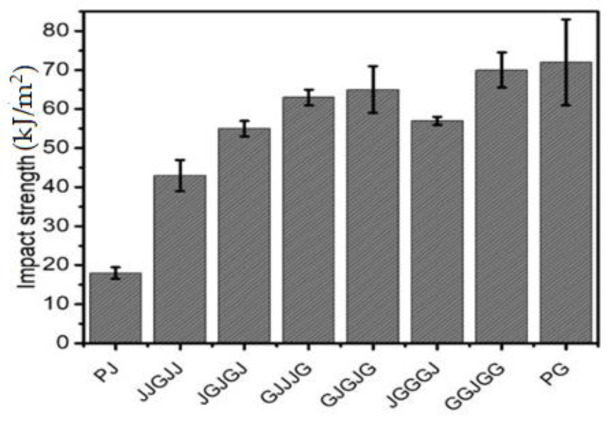
Impact strength of different samples.

Impact is a significant phenomenon in the regulation of a structure’s life. Impact experiments were used to study material toughness. A material’s toughness is an indicator of its ability to absorb energy. during plastic deformation. The impact strength values of the jute fiber, glass fiber composites, and hybrid composites of various stacking arrangements are presented in Table 3 and Figure 5. The jute composite’s impact strength value was lower than that of the other composites, with a value of 18 kJ/m^2^. The reason was that the jute fiber’s strength and stiffness were lower, resulting in a reduced ability to absorb impact energy. In comparison, the glass fiber composite showed a maximum impact strength of 72 kJ/m^2^. This was due to its high resistance to stress propagation. The inclusion of glass fiber with jute fiber in the hybrid composites led to a significant improvement in impact strength. By comparing hybrid composites with the same layers of jute/glass and distinct stacking combinations, e.g., GJGJG with JGGGJ and GJJJG with JGJGJ, it was observed that the percentage increase in impact strength of GJJJG and GJGJG was 15% and 14% higher than JGJGJ and JGGGJ, respectively. These results were consistent with De Rosa et al. [33], who explored hybrid composites enclosing glass/jute/glass plies and disclosed that the failure initially occurred on the top of the composite and that, consequently, the plies further off the core experienced the highest loss. Comparing GJJJG and JGGGJ, it was apparent that composites with glass plies positioned farther from the core had a high impact resistance [34]. It was also suggested that glass fibers’ positioning on the surface could enhance the composite’s bending stiffness and increase the impact strength [18].

### 3.4. Morphological Analysis

**Figure 6:**
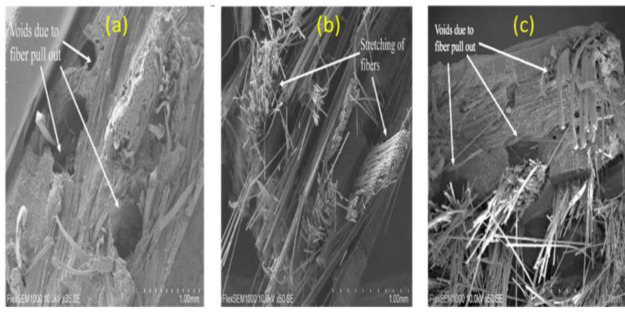
SEM images of (**a**) jute fiber composite, (**b**) glass fiber composite and (**c**) hybrid composite (scale bar 1.0 mm).

The pure and hybrid composites’ tensile fractured specimens were observed through a scanning electron microscope to assess their morphological structures. The SEM micrographs of jute, glass, and hybrid composites of jute/glass used in this study are shown in Figure 6, illustrating the reasons and types of failure for the pure and hybrid composites during tensile testing. Fiber fracture, fiber pull out, and voids on the fractured surface of the composites were observed. The voids in Figure 6a were evident on the surface of the jute fiber composite, suggesting weak interfacial adhesion of the fiber and matrix. This could suggest that fiber was easily removed from the interfacial region through weak compatibility, causing the composite to collapse rapidly. Glass fiber stretching and elongation are shown in Figure 6b; this occurred as a result of the tensile strength applied. The extension of the fiber showed that the polymer’s strength was improved because of the introduction of glass fiber in the PBS composite. There was extensive fiber pullout or breakage in the hybrid configurations, as shown in Figure 6c. 

Another explanation for this may be that the glass fibers (2.5–3.0) were more extensible than the jute fibers (1.5–1.8) [35]. A composite’s tensile properties depend mostly on the elongation at break percentage and the single-fiber modulus for hybrid composites. Delamination was found in the jute and glass hybrid composites at the interface after they were mounted in tension, as had been observed in the failed specimens.

### 3.5. Water Absorption and Thickness Swelling

**Figure 7:**
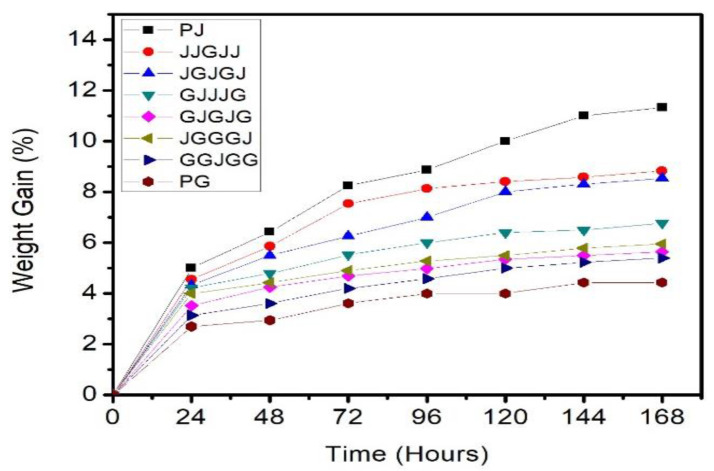
Water absorption of specimens.

Water absorption is a valuable property and was used to estimate PBS composites’ performance reinforced by jute/glass fiber. Figure 7 illustrates the measured values of jute composite, glass composite, and hybrid composites of jute/glass with different stacking sequences by the water absorption test. 

It was seen that the jute fiber composite absorbed more water than the other composites. The jute fiber composites’ hydrophilicity resulted in higher water absorption due to the lignin and hemicellulose components. Hemicelluloses were primarily accountable for water absorption, while non-crystalline cellulose and lignin played a significant part in the procedure. Water caused the cell wall of the fiber to enlarge until it saturated. It continued to fill the fiber’s gap spaces, and this free water did not cause additional swelling. 

On the other hand, the pure glass composite absorbed the lowest quantity of water in comparison to the other composites. The introduction of glass fibers to the jute/PBS composite had a substantial influence on decreasing the water absorption of the sample. By integrating glass fibers, the jute fiber composite water absorption was significantly enhanced. It could be established that higher fiber loadings of natural fibers lead to higher absorption of water. By contrast, increases in the loading of glass fiber within hybrid composites result in lower water and moisture absorption, with reduced hydrophilic jute fiber content and increased hydrophobic glass fiber content. This observation is compatible with previous work by Kushwaha [36], who found that the introduction of glass fiber decreased the absorption of water through epoxy as well as polyester bamboo composites. With the application of only one ply of glass fiber, the water absorption capacity of the jute fiber composite decreased by 36.71%. The ideal design for hybrid composites of natural/synthetic fiber was to place the synthetic fibers in an extreme position to ensure minimal moisture absorption. The reason behind this was that natural fibers are hygroscopic. When synthetic fiber was mounted on the composite’s outer surface, the sample’s hygroscopicity was improved by the protection created by the more impermeable synthetic fibers.

**Figure 8:**
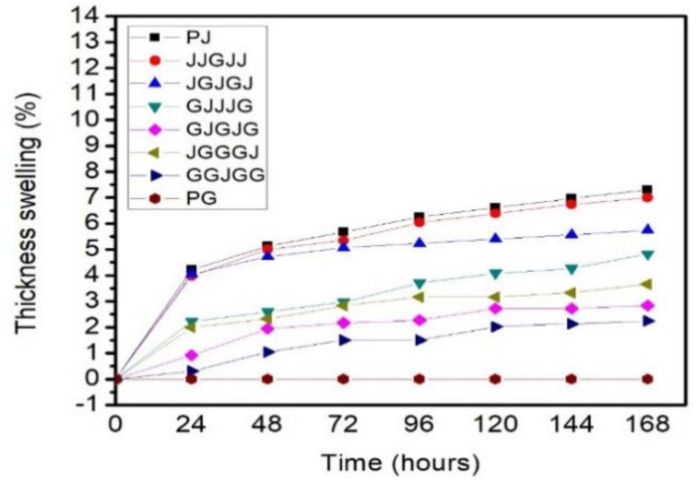
Thickness swelling of specimens.

Figure 8 displays the values of thickness swelling attained after seven days of immersion of composites. The pure jute composites showed a higher thickness swelling value of 7.3% relative to the other composites for long-term immersion. However, the pure glass composite did not show any change, even after a 168 hours immersion time. It was revealed that the lower thickness swelling trend value was due to the higher glass fiber content in the PBS composites. Regarding the hybridization of jute/glass, the GGJGG displayed the lower thickness swelling, followed by GGJGG < GJGJG < JGGGJ < GJJJG < JGJGJ < JJGJJ composites. This suggested that replacing jute fiber layers with other glass fibers decreased the thickness and swelling properties, although the jute fiber content in the hybrid formulation was reduced.

### 3.6. Thermal Studies

TGA is a useful technique for measuring the change in weight with temperature to quantitatively conclude how the fibers and matrix in a composite degrade and how they assemble. The consideration of thermal stability is quite relevant for jute composites and hybrids for use in load-bearing applications. The TGA results demonstrated that the jute composites’ thermal stability was significantly affected by glass fiber hybridization. 

For the jute, glass, and jute/glass-based PBS hybrid composites, degradation was carried out using thermo-gravimetric (TGA) The decomposition rate at the peak and the non-volatile content at 600 °C (expressed in char) are summarized in Table 4. The decomposition temperature of the glass fiber sample was 326 °C. It was found that the matrix and the different reinforcement components in the composite influenced the composite’s thermal stability. Small degradation peaks were seen from 230 °C and 330 °C due to the decomposition of the composites’ organic elements. The second stage, from about 330 °C to 360 °C, was attributed to the breakdown of the hemicellulose components in the jute fiber, and the third peak at 420 °C represents the degradation of the PBS resin. The composites’ thermal degradation increased below about 420 °C, while it decreased to above 420 °C. In general, the PBS in the lower temperature range accounted for the lower thermal stability of the jute fabric; however, the lower thermal stability of the PBS in the high-temperature range was estimated for the inclusion of jute fabric. Consequently, the reinforcement of jute fabric in the PBS could serve as an obstacle to improved heat insulation and prevent the embedding of volatile degradation materials in composites. 

**Figure 9:**
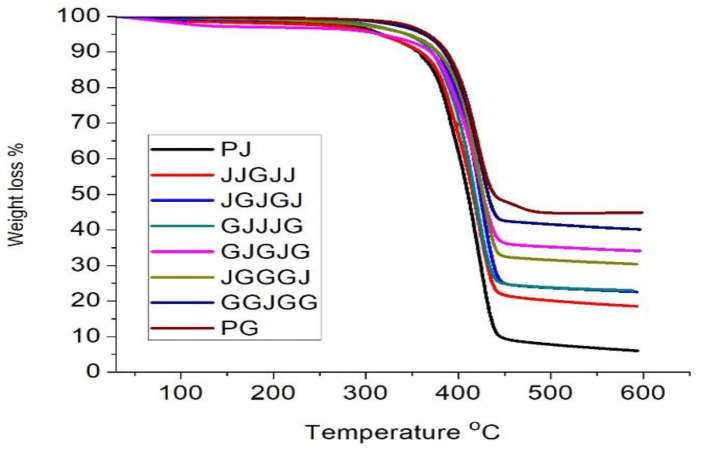
TGA curves of different samples.

The TGA results of the pure glass fiber reinforced with PBS are shown in Figure 9. The composite started to lose only 0.1% of its original weight until after the temperature reached 100 °C; up to 200 °C, the loss of weight was already 0.23% of the weight, corresponding to the elimination of moisture in the matrix. At 200 °C and 450 °C, the weight loss was nearly 46% due to decomposition. The volatilization of the matrix in the composite then retained a natural weight loss up to 600 °C, where only 54% of the original weight constituted the actual remainder.

The composite with more glass fibers showed a change in the process of degradation at a higher heating rate of about 435 °C, as per the TGA curve in Figure 9. As shown in Table 4, the highest temperature around the higher weight loss (T-max) improved with the loading of the glass fibers, indicating that the addition of glass fibers improved the jute/glass fiber hybrid PBS composite’s thermal stability. An identical result was obtained with the thermal degradation of the jute/glass fiber hybrid composites. Furthermore, early decomposition temperature was observed at the higher fiber content of jute fiber relative to only glass fiber composites. From these results, it can be inferred that the integration of jute fiber decreased the hybrid composite’s degradation temperature [37].

Additionally, hybrid composites of jute/glass containing more jute fiber plies showed more significant weight loss (low residue of char) concerning composites with more glass plies. Jute fiber could be regarded as significantly involved in improving the residue of hybrid jute/glass composites. The introduction of higher glass fiber content in the PBS matrix reinforced with jute fiber led to an increase in the onset and maximum composite decomposition temperature.

## 4. Conclusions

An experimental study was carried out on the influence of stacking sequences on the mechanical, water absorption, and thermal properties of jute/glass-fiber-reinforced PBS composites. The following results were obtained.

(1) The pure jute composites showed the lowest mechanical properties because of the lower strength of the jute fiber, the presence of voids, and poor adhesion with the matrix.
(2) The hybrid GJ composites showed optimum mechanical properties in comparison to the pure composites. The hybrid composites performed better in tensile properties with the addition of glass layers in the jute composites. in other words, the enhancement of the tensile properties of jute composites depends on the amount of hybridized or sandwiched glass composites.
(3) The hybrid composites’ enhanced flexural property showed that the flexural strength improved with the glass fiber content, particularly for hybrid composites enclosing glass fabric plies at the outer layers. This was due to the fact that the top layer of the reinforcement layer controlled the flexural strength and stiffness of the specimen.
(4) Impact studies showed that glass fiber layers should be positioned on the surface of the composite in order to ensure optimum results, an effect that is even more significant than increasing the number of glass fiber plies, as found by the comparing JGGGJ and GJJJG specimens.
(5) The SEM analysis of the tensile fractured samples showed that the lower strength of the jute fiber composite was due to the poor adhesion and fiber pullout, whereas the higher strength of the glass fiber composites was due to better interfacial properties. Extensive fiber pullout and fiber breakage were observed for the hybrid composites.
(6) Adding glass fiber led to a decrease in the water absorption and thickness swelling of the jute composites because the glass fiber’s outer plies acted as barriers shielding the inner plies from direct contact with the water.
(7) The thermal stability of the hybrid composites was enhanced because the initial and final decomposition temperatures of the hybrid composites moved to higher temperatures through glass fiber hybridization.

Furthermore, the above-mentioned mechanical results were found to be critical in determining the quantity of glass fiber, which may be replaced with jute fiber in order to customize the final composite’s qualities. Further research should be carried out to check the effect at different strain rates or loading rates of, for example, creep and stress relaxation, quasi static loading, low impact loading, high impact loading, on this type of hybrid composite.

## Figures and Tables

**Table 1:** Jute and glass fabric properties.

Fabric	Woven Type	Thickness (mm)	Thread Counts per 100 mm	
			Warp	Weft
Jute	Plain	0.7	37	37
Glass	Plain	0.2	75	80

**Table 2:** The volume fraction of each constituent.

Layering Sequence	Jute Fiber Volume Fraction (%)	Glass Fiber Volume Fraction (%)
PJ	100	0
JJGJJ	80	20
JGJGJ	60	40
GJJJG	60	40
JGGGJ	40	60
GJGJG	40	60
GGJGG	20	80
PG	0	100

PJ = Pure jute fabric layer; PG = Pure glass fabric layer.

**Table 3:** Mechanical properties of jute fiber, glass fiber, and hybrid composites.

Groups	Tensile Strength (MPa)	Tensile Modulus (MPa)	Flexural Strength (MPa)	Flexural Modulus (MPa)	Impact Strength (kJ/m^2^)
PJ	39 ± 2.0	2313 ± 54.0	37 ± 1.5	701 ± 27	18 ± 1.5
JJGJJ	65 ± 2.0	2975 ± 126	41 ± 1.5	862 ± 41	43 ± 4.0
JGJGJ	105 ± 2.0	3431 ± 167	44 ± 1.5	1024 ± 110	54 ± 2.0
GJJJG	118 ± 5.0	4113 ± 247	51 ± 3.0	1348 ± 189	57 ± 1.0
GJGJG	145 ± 4.0	4619 ± 87.0	55 ± 2.0	1600 ± 66	65 ± 6.0
JGGGJ	113 ± 7.0	3854 ± 266	47 ± 1.0	1293 ± 117	63 ± 2.0
GGJGG	154 ± 6.0	5250 ± 97.0	60 ± 1.0	1746 ± 52	70.5 ± 4.0
PG	216 ± 10.0	6601 ± 206	64 ± 2.0	2543 ± 125	72 ± 11.0

PJ = Pure jute fabric layer; PG = pure glass fabric layer.

**Table 4:** TGA analysis for jute/glass-reinforced PBS hybrid composites.

Groups	T ON (°C)	T Max (°C)	Weight Loss (wt. %)	Char at 600 °C (wt. %)
PJ	378	437	94	6
JJGJJ	378	437	82	18
JGJGJ	389	438	78	22
GJJJG	389	439	77	23
JGGGJ	384	439	71	29
GJGJG	390	439	66	34
GGJGG	390	439	60	40
PG	391	439	54	46

PJ = Pure jute fabric layer; PG = pure glass fabric layer.

## Data Availability

Not applicable.
